# Effects of *Lippia sidoides* essential oil, thymol, p-cymene, myrcene and caryophyllene on rat sciatic nerve excitability

**DOI:** 10.1590/1414-431X20176351

**Published:** 2017-10-19

**Authors:** R. Barbosa, Y. Cruz-Mendes, K.S. Silva-Alves, F.W. Ferreira-da-Silva, N.M. Ribeiro, L.P. Morais, J.H. Leal-Cardoso

**Affiliations:** 1 Laboratório de Eletrofisiologia, Instituto Superior de Ciências Biomédicas, Universidade Estadual do Ceará, Fortaleza, CE, Brasil; 2 Laboratório de Fisiofarmacologia das Células Excitáveis, Universidade Regional do Cariri, Crato, CE, Brasil

**Keywords:** Compound action potential, Essential oil, Lippia sidoides, Thyme oil, p-cymene, Myrcene

## Abstract

*Lippia sidoides* Cham is a typical herb species of Northeast Brazil with widespread use in folk medicine. The major constituents of the essential oil of *L. sidoides* (EOLs) are thymol, p-cymene, myrcene, and caryophyllene. Several studies have shown that the EOLs and its constituents have pharmacological effects, including antibacterial, anti-inflammatory, antioxidant and neuroprotective activity. Therefore, this work aimed to investigate the effects of the EOLs and their main constituents on rat sciatic nerve excitability. The sciatic nerves of adult Wistar rats were dissected and mounted in a moist chamber. Nerves were stimulated by square wave pulses, with an amplitude of 40 V, duration of 100 μs to 0.2 Hz. Both EOLs and thymol inhibited compound action potential (CAP) in a concentration-dependent manner. Half maximal inhibitory concentration for CAP peak-to-peak amplitude blockade were 67.85 and 40 µg/mL for EOLs and thymol, respectively. CAP peak-to-peak amplitude was significantly reduced by concentrations ≥60 µg/mL for EOLs and ≥30 µg/mL for thymol. EOLs and thymol in the concentration of 60 µg/mL significantly increased chronaxie and rheobase. The conduction velocities of 1st and 2nd CAP components were also concentration-dependently reduced by EOLs and thymol in the range of 30-100 µg/mL. Differently from EOLs and thymol, p-cymene, myrcene and caryophyllene did not reduce CAP in the higher concentrations of 10 mM. These data demonstrated that EOLs and thymol inhibited neuronal excitability and were promising agents for the development of new drugs for therapeutic use.

## Introduction

*Lippia sidoides* is a common herb species from Northeast Brazil, used as a topical anti-septic agent for skin, mucous and throat infection (1). The essential oil of *Lippia sidoides* (EOLs) inhibits fungi growth of *Microsporum canis*, *Microsporum* and *Candida* spp pachydermatis species and recent studies demonstrate that this oil also inhibits the growth of *Leishmania chagasi* (1,2).

The main constituents of EOLs are the monoterpenoids thymol, p-cymene and myrcene, and the sesquiterpene caryophyllene (1). Several studies show that thymol inhibits antibacterial and antifungal growth, and has been used for oral hygiene due to reduction of microbacterial growth (3). This molecule also acts on membrane receptors and ion channels. Thymol activates human transient receptor potential family Ankyrin subtype 1 (hTRPA1) and modulates the thermo-transient receptor potential (thermo-TPP) (4,5). It was also documented that thymol inhibits voltage-dependent sodium channel subtypes expressed in cells HEK-293 (6). The other constituents of EOLs, myrcene, caryophyllene and p-cymene are reported to have antimicrobial (7,8), anti-inflammatory (9), and neuroprotective effects (10). Furthermore, caryophyllene also has an analgesic effect (11).

It was demonstrated that EOLs possess relevant biological properties (1–3) and are thought to be a source of potent biological compounds, since several constituents of essentials oils show pharmacological activity, including blocking nerve excitability and acting on Na^+^ channels, such as eugenol, linalool, carvacrol, estragole, and cineole (12–16). However, there is no description of the effect of EOLs thymol, p-cymene, myrcene and caryophyllene on sciatic nerve excitability, which was demonstrated by other terpenes and terpenoids. Thus, this work had the objective to study the action of EOLs thymol, p-cymene, myrcene and caryophyllene on rat sciatic nerve excitability

## Material and Methods

### Extraction and chemical analysis

EOLs was provided and prepared by Dr. Sergio Horta. EOLs was analyzed at Technological Development Park (PADETEC) of the Universidade Federal do Ceará (UFC). EOLs analysis was performed on a Hewlett-Packard 6971 gas chromatography and mass spectrometry (GC/MS, USA). Briefly, we used a dimethylpolysiloxane DB-1 fused silica capillary column (30 m × 0.25 mm; 0.1 µm), helium carrier gas (1 mL/min), injector temperature of 250°C, detector temperature of 200°C, column temperature of 35-180°C at 4°C/min, and then 180-250°C at 10°C/min, mass spectra of electronic impact 70 eV. The composition of EOLs determined by this method were: thymol (66.00%), p-Cymene (15.01%), caryophyllene (4.60%), myrcene (4.23%), gamma-terpinene (1.87%), thymyl-ethyl-ether (1.15%), and alfa-terpinene (1.01 %). They were identified using a mass spectral library search and 13C-NMR spectroscopy.

### Animals

Wistar rats (200–250g) of both sexes were kept under constant temperature (22±2°C) with a 12-h light/dark cycle and free access to food and water. All animals were handled in compliance with the Guide for the Care and Use of Laboratory Animals, published by the US National Institutes of Health (NIH Publication 27-89, revised 1996; http://www.nap.edu), and all efforts were made to minimize animal suffering. Procedures described herein were first reviewed and approved by the local animal ethics committee (CEUA/UECE - Protocol No. 10725297-0).

### Solutions and reagents

Locke's solution was used for extracellular recording, of which composition was: 140 mM NaCl, 5.6 mM KCl, 1.2 mM MgCl_2_, 2.2 mM CaCl_2_, 10 mM tris(hydroxymethyl-aminomethane), and 10 mM glucose, pH adjusted to 7.4, with HCl. Due to low solubility of EOLs thymol and p-cymene in water, they were dissolved in dimethyl sulfoxide (DMSO), in which the final concentration never exceeded 0.2% v/v. Stock solutions of EOLs and its constituents were prepared daily, diluted in Locke's solution for desired concentration and added to the chamber for extracellular recordings. The concentrations of EOLs and thymol used were 10, 30, 60, and 100 μg/mL, and 200 μg/mL for EOLs only. P-cymene, myrcene and caryophyllene were used in unique concentrations of 1.79, 2.04, and 1.36 mg/mL, respectively. Experiments were carried out at room temperature (22 to 26°C). Thymol, p-cymene and other reagents were of analytical grade and were purchased from Sigma Chemical (USA).

### Extracellular recording

The sciatic nerve was stimulated, and evoked compound action potential (CAP) was recorded as described by Lima-Accioly et al. (17). The sciatic nerve was dissected from rats sacrificed by carbon dioxide inhalation. The nerve was immediately placed in a Petri dish containing ice cold (4°C) modified Locke's solution and they were used for experimental recording in the same day. The sciatic nerve was mounted in a moist chamber and one of its ends was stimulated with a stimulus isolation unit connected to a stimulator to evoke compound action potential (Model S48, Grass Instruments Co., USA). Evoked signal was recorded with electrodes placed 40 to 50 mm from the stimulating electrodes and monitored using an oscilloscope (Model 547, Tektronix, Inc., USA) and continuously stored in personal computer by acquisition software/hardware system (pClamp 9/Digidata 1332A; Molecular Devices, USA) for further analysis. To administer EOL and its constituents and maintain chamber humidity, the sciatic nerve (15 to 20 mm) was suspended between the stimuli and recording electrodes but a segment of the nerve was immersed in a layer of Locke's solution maintained at the bottom of the chamber. EOL, thymol, p-cymene, myrcene and caryophyllene exposition was initiated only when stable CAP peak-to-peak amplitude was achieved for at least 30 min, and exposure time lasted 180 min. This period was followed by a washout recovery period of 180 min. Electrophysiological parameters measured in extracellular recording were rheobase, chronaxie, peak-to-peak amplitude (PPA), and conduction velocity of CAP components.

### Statistical analysis

Results are reported as means±SE, including for half maximal inhibitory concentration (IC50) values. In some cases, IC50 values are reported as means±SE (n), for which “n” indicates the number of experiments. The unpaired Student's *t*-test and one-way ANOVA followed by the Tukey multiple comparison test were used when appropriate. Two means were considered to be statistically different when P<0.05.

## Results

**Figure 1.**
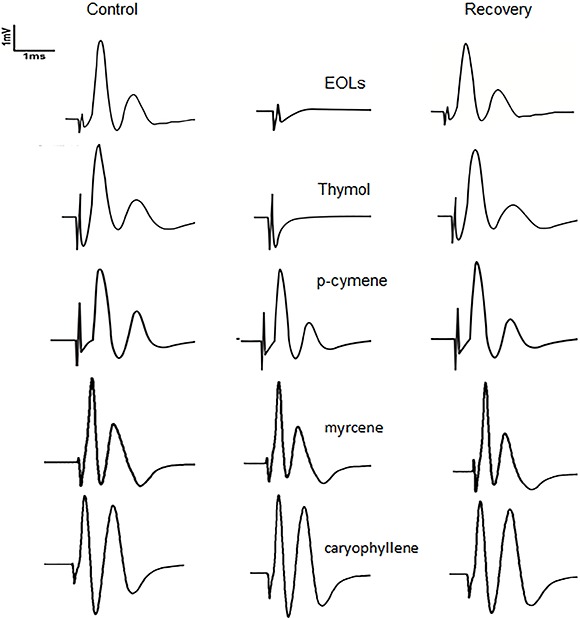
Compound action potential (CAP) inhibition of rat sciatic nerve promoted by essential oil of *L. sidoides* (EOLs), thymol and p-cymene. Illustrative CAP traces are shown in control, at the end of 180 min exposure to EOLs (200 µg/mL), thymol (100 µg/mL), p-cymene, caryophyllene and myrcene (10 mM) and after 180 min of washout (recovery).

**Figure 2.**
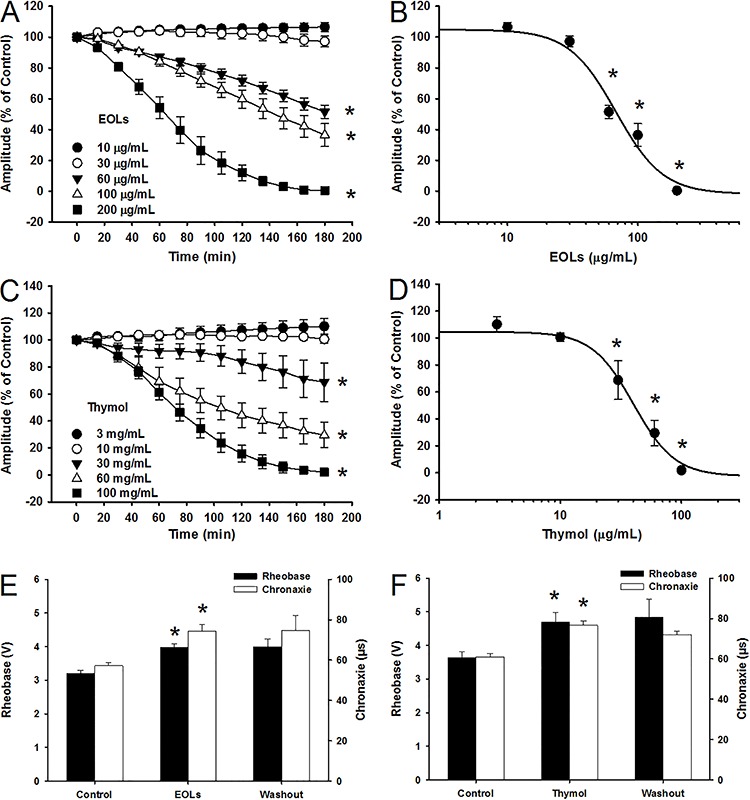
Amplitudes and excitability parameters of compound action potential. Panels A and C present the time course of essential oil of *L. sidoides* (EOLs) and thymol effects on peak-to-peak amplitude, and panels B and D show concentration-response curves for EOLs and thymol, respectively. Panels E and F show effects of EOLs (60 μg/mL) and thymol (60 μg/mL; 0.4 mM) on rheobase and chronaxie. Data are reported as means±SD. *P<0.05 compared to control (ANOVA followed by Bonferroni's *post hoc* test).

EOLs and thymol, in the concentration of 3–200 µg/mL (Thymol, 100 µg/mL=0.6 mM), reversibly blocked CAP whilst p-cymene, caryophyllene and myrcene in 10 mM did not affect CAP (Figures 1 and 2).

CAP blockade by EOLs was concentration-dependent (Figure 2A and B). At the end of 180 min exposure to 10 and 30 µg/mL EOLs (n=6), there was no significant reduction (P>0.05) in CAP PPA. At the end of 180 min exposure to 60, 100, and 200 µg/mL, there was a reduction in CAP PPA of 55.5, 45.8, and 0.4%, respectively, compared to control (P<0.05).

A similar effect was found for thymol (Figure 2C and D). There was no difference between thymol and EOLs, concerning pharmacological potency (Figure 2B and D), for the effect on CAP PPA. Regarding time course (Figure 2C), at the end of 180 min exposure to 30, 60, and 100 µg/mL thymol (n=6), PPA was significantly reduced to 68.73±14.29, 29.57±9.36 and 1.94±1.94%, respectively (P<0.05, ANOVA); with 3 and 10 µg/mL, there was no significant effect compared to control (P>0.05).

Concerning parameters related to excitability, the rheobase and chronaxie values of control conditions were 3.4±0.1 V and 58.8±1.5 µs (n=6; Figure 2E). In the presence of EOLs, there was a significant increase in both parameters and the experimental values were 3.9±0.1 V and 74.2±4.2 µs, respectively. For thymol, these two parameters were increased to 4.8±0.2 V and 77.2±2.4 µs (n=6), respectively (Figure 2F).

**Figure 3.**
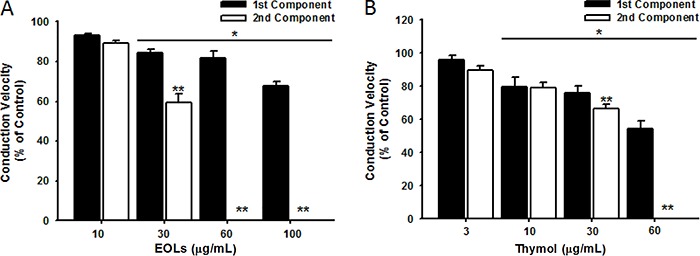
Effect of essential oil of *L. sidoides* (EOL) and thymol on conduction velocity of compound action potential component. Data are reported as means±SD. *P<0.05, **P<0.01, compared to control (ANOVA, followed by Bonferroni's *post hoc* test).

The conduction velocities of both CAP components were also progressively reduced after exposure to EOLs and thymol. At the end of 180 min exposure to 10, 30, 60, and 100 µg/mL EOLs, the conduction velocity of the 1st component was significantly (P< 0.05) reduced to 93.2, 84.5, 81.8, and 67.5 (n=6) of control, respectively (Figure 3A); for 10, 30, and 60 µg/mL thymol, the reductions were 79.6, 75.94, and 54.5% of control, respectively (Figure 3B). Concerning the 2nd component, exposure to 10 and 30 µg/mL EOLs significantly (P<0.05) reduced the conduction velocity to 89.2 and 59.1% of control, and 3, 10, and 30 µg/mL thymol reduced to 89.9, 79.0, and 66.7% of control, respectively (Figure 3B). At concentrations greater than 60 µg/mL, the reduction on PPA did not permit calculation of velocity conduction (Figure 3A and B). All EOLs and thymol effects developed slowly and were reversible after 300 min of washout.

## Discussion

This work demonstrated that EOLs and its main constituent thymol (percentage: ∼66% of oil sample) are effective depressors of neuronal excitability. This effect was shown by the experimental analysis that quantified several electrophysiological parameters of sciatic nerve CAP, such as PPA, conduction velocity, rheobase and chronaxie. It was also verified that thymol was likely to be pharmacologically more potent than EOLs, since thymol IC50 was smaller than EOLs for PPA, (40 *vs* 67.85 µg/mL, respectively), and 3 µg/mL of thymol, but not EOLs, significantly decreased first component conduction velocity.

In contrast, it was demonstrated in this study that p-cymene, caryophyllene and myrcene, even as important constituents in EOLs samples (∼15, 4.60 and 4.23%), did not promote a significant alteration in electrophysiological parameters of sciatic nerve CAP. These results suggest that CAP blockade by EOLs could be attributed solely to the presence of thymol, considering that p-cymene, and caryophyllene and myrcene presented no effect and the other constituents are in minor proportions in the oil samples.

EOLs and thymol may be acting on the sciatic nerve voltage-dependent sodium channel, but more study is necessary. These results also indicate that EOLs and thymol very likely possess a local anesthetic activity and its effects resemble other constituents such as linalool, carvacrol, estragole and cineole (13–16).

The CAP wave exhibited the presence of two components according to conduction velocity (14,18). Considering that conduction velocity of nerve impulse is proportional to the fiber diameter, fibers with great diameter have higher velocities and small diameter fibers have lower conduction velocity (19). Therefore, one can classify the fiber types present in each component of CAP according to the conduction velocity.

Data regarding conduction velocity showed a mean value of 78.11±1.84 and 27.53±0.69 m/s for 1st and 2nd CAP components, respectively. These velocities can be categorized in myelinated Aα and Aβ motor fibers for 1st component and myelinated motor and sensorial Aγ and Aδ fibers for 2nd component. Concerning the effects of EOLs and thymol, these substances inhibited the amplitudes and conduction velocities of both CAP components and the effect was more pronounced in the 2nd component, i.e., in sensorial and motor myelinated fibers of small diameter.

The changes that EOLs and thymol made on rheobase and chronaxie (parameters more directly related to nerve excitability) show that they reduce neuronal excitability, which might indicate a possible potential for local anesthesia use. Our data corroborate the literature about antinociceptive activity (20) since local anesthesia could be considered a mechanism to interrupt pain through the blockade of action potential (12).

In conclusion, this study shows that EOLs and thymol blocked nerve excitability. This result can contribute to a better understanding of the action of essential oils and their constituents upon electrical activity of neuronal cells. Blockade of the excitability of peripheral nerves is very probably due to a local anesthetic mechanism. Thus, EOLs and thymol may be potentially useful for therapeutic use.
